# Reconceiving representation-hungry cognition: an ecological-enactive proposal

**DOI:** 10.1177/1059712318772778

**Published:** 2018-05-23

**Authors:** Julian D Kiverstein, Erik Rietveld

**Affiliations:** University of Amsterdam, Amsterdam, The Netherlands

**Keywords:** Enaction, representation-hungry problems, affordances, biological autonomy, skilled Intentionality, episodic memory imagining, micro-identities

## Abstract

Enactive approaches to cognitive science aim to explain human cognitive processes across the board without making any appeal to internal, content-carrying representational states. A challenge to such a research programme in cognitive science that immediately arises is how to explain cognition in so-called ‘representation-hungry’ domains. Examples of representation-hungry domains include imagination, memory, planning and language use in which the agent is engaged in thinking about something that may be absent, possible or abstract. The challenge is to explain how someone could think about things that are not concretely present in their environment other than by means of an internal mental representation. We call this the ‘Representation-Hungry Challenge’ (RHC). The challenge we take up in this article is to show how hunger for representations could possibly be satisfied by means other than the construction and manipulation of internal representational states. We meet this challenge by developing a theoretical framework that integrates key ideas drawn from enactive cognitive science and ecological psychology. One of our main aims is thus to show how ecological and enactive theories as non-representational and non-computational approaches to cognitive science might work together. From enactive cognitive science, we borrow the thesis of the strict continuity of lower and higher cognition. We develop this thesis to argue against any sharp conceptual distinction between higher and lower cognition based on representation-hunger. From ecological psychology, we draw upon our earlier work on the rich landscape of affordances. We propose thinking of so-called representation-hungry cognition in terms of temporally extended activities in which the agent skilfully coordinates to a richly structured landscape of affordances. In our framework, putative cases of representation-hungry cognition are explained by abilities to coordinate nested activities to an environment structured by interrelated socio-material practices. The RHC has often figured in arguments for the limitations of non-representational approaches to cognitive science. We showcase the theoretical resources available to an integrated ecological-enactive approach for addressing this type of sceptical challenge.

## 1. Introduction

Every science operates with theoretical assumptions that guide scientific research, shaping how scientists think about the phenomena they investigate, framing the research questions scientists ask (Fleck, 1979; Kuhn, 1962). Since its birth in the 1950s, cognitive science has drawn its theoretical assumptions from the computer theory of the mind. According to the computer theory of mind, cognitive processes are mechanistically realised in computational processes of building, storing and manipulating content-bearing internal representations. While there has always been much debate about the nature of mental representation, it has been something of a near consensus among cognitive scientists that cognitive processes must involve in some way processes of building and manipulating internal representational states. In the last 25 years, this consensus has begun to unravel. Research programmes have begun to emerge from the margins of cognitive science that reject either in part or in whole the computer theory of mind. But every science needs a theoretical framework. This is where the enactive approach to cognitive science enters the story (Di Paolo, Rohde, & De Jaegher, 2010; Thompson, 2007; Varela, Thompson, & Rosch, 1991). The enactive theorists propose a new set of theoretical assumptions for understanding what cognition is and how it works that aims to break the tight conceptual connection between cognition and representation.

A challenge to the enactive research programme that immediately arises is how to explain cognition in so-called ‘representation-hungry’ domains (Clark & Toribio, 1994).^1^ Examples of representation-hungry domains include imagination, memory, planning and language processing. These are examples of cognitive activities in which an agent can be engaged in thinking about something that is absent, counterfactual or abstract. When one remembers some past experience, say the house in which one grew up, one is recalling an experience of a place in which one is no longer present, and may not have been present for quite some time. When one imagines what it might be like to visit a country on holiday, what one imagines is an event that might possibly take place in the future, and may well never occur. Finally, many of the properties that people can categorise and think about involve the tracking of patterns and regularities that cannot be concretely observed and that instead take the form of complex and unruly similarities and differences. We can, for instance, think about all of the objects that belong to the Pope (to offer an example discussed by Clark and Toribio (1994)). There is presumably very little that such objects share in common at the level of their concrete, observable properties other than that they belong to one and the same man. The challenge is to explain how someone could entertain thoughts like these without making use of internal representational states that target objects and their properties not currently present in the thinker’s environment.

In what follows, we will use the term ‘representation’ to denote states of a system that stand in and function as surrogates for things in their absence (Clark & Grush, 1999; Haugeland, 1991; Orlandi, 2014; Ramsey, 2007). Any state of a system that has the function of standing in for *x* can also fail to perform this function. I can, for instance, misremember some feature of the house in which I grew up as a child: I might think the walls were painted blue when they were in fact painted green. Thus, we can think of states that function as stand-ins as states that take the world to be a certain way. Whether the world is the way it is taken to be is a further question. Thus, states that function as stand-ins for external states of affairs can be assessed for correctness or incorrectness, truth or falsity, accuracy or inaccuracy, appropriateness or inappropriateness and so on.

Cognitive processes such as imagination, memory and abstract thought might intuitively strike one as being representation-hungry. It seems just obvious that the only way these processes could possibly work is through the mediation of internal representational states. It is natural to think that whenever I think about an object or property *x* that is absent, counterfactual or abstract, I can do so only by occupying a state that has the function of standing in for *x*. After all, the thing in the world my thoughts target is not there, and might even never exist. How else could I entertain thoughts about that thing if not by means of having an internal state that functions as a stand-in for that thing? The challenge we take up in this article is to show how hunger for representations could possibly be satisfied by means other than the construction and manipulation of internal representational states. We will henceforth refer to this challenge as the ‘Representation-Hungry Challenge’ (RHC). We will also often talk of ‘representation-hungry cognitive processes’, though if our argument succeeds it will turn out that properly speaking there are no representation-hungry cognitive processes.^2^

One quick and dirty solution available to enactivists would be to make a sharp conceptual distinction between higher and lower levels of cognition. Enactive theories might then be argued to be restricted in their scope to cases of cognition in which ‘the world can serve as its own best model’ (Brooks, 1991). In this strategy for meeting RHC, enactive-style explanations will be reserved for lower-level, online sensorimotor forms of control and engagement with the environment. All cases of higher-level cognition by contrast would call for non-enactive styles of explanation in terms of offline and fully decoupled processing of internal mental representations. Such a solution would however require justifying a sharp conceptual distinction between higher and lower cognition, or equivalently between online and offline cognition.^3^

In what follows, we defend a strict continuity of higher and lower levels of cognition. The notion of ‘strict continuity’ we have in mind here is consistent with there being important differences between higher and lower varieties of cognition. We take strict continuity to imply however that higher forms of cognition are elaborations and gradual complexifications that develop out of lower, non-representational forms of cognition. We are thus committed to the controversial hypothesis that at no point in the natural history of human minds did our ancestors find it necessary to construct, store and manipulate *internal* mental representations.^4^ Any watering-down or restriction of the scope of enaction to cases of online sensorimotor control is unwarranted and unnecessary. It is unwarranted because no sharp conceptual distinction between higher and lower cognition can be defended. It is unnecessary because the concept of enaction can scale up to account for higher cognition.

To defend the strict continuity of lower and higher cognition will however depend upon recognising the contribution of the surrounding environment to cognition. What is distinctive about higher cognition is its temporal reach. The abilities that are characteristic of higher cognition allow the agent to engage with possibilities that are increasingly remote in space and time. We will argue that to do justice to this point will require the enactive theory to do justice to the role of the environment in guiding and constraining cognitive processes. This is something enactivists have often gestured at in their writings without satisfactorily explaining.^5^ By contrast, ecological psychologists have dedicated themselves to investigating this very phenomenon. We will therefore propose a synthesis of ecological and enactive approaches to cognitive science. Both have provided non-representational conceptual frameworks for the explanation of human cognition. We will show how these conceptual frameworks can profitably be combined. The result is a non-representational theoretical framework for cognitive science that has the explanatory power to address the RHC.

Representation-hungry cognition is standardly thought of as a matter of inferential reasoning taking place inside of the heads of individuals. Such inferential reasoning is detached and decoupled from the world insofar as it is carried out on states that function as surrogates for distal, abstract or counterfactual states of affairs. We will propose thinking of so-called representation-hungry cognition in terms of temporally extended activities in which the agent skilfully coordinates to a richly structured landscape of affordances (Bruineberg & Rietveld, 2014; Rietveld & Kiverstein, 2014; Van Dijk & Rietveld, 2016; c.f. Van Dijk & Withagen, 2016). Putative cases of representation-hungry cognition can thus be explained by abilities to coordinate nested activities to an environment structured by interrelated socio-material practices.^6^

In making such a proposal, we do not claim to have adequately addressed every dimension of the RHC. Our aim in this article is primarily to provide an alternative conceptual framework for thinking about representation-hungry cases. We set out to show how agents can do without representations in domains of thinking that have traditionally been thought to be fuelled by internal representations. The RHC has figured in arguments for the limits of explanations in the cognitive sciences that try to do without the concept of mental representation (see, e.g., Clark, 2001; Shapiro, 2011; Wheeler, 2005). We showcase the theoretical resources available to a combined ecological and enactive approach for addressing this type of sceptical challenge.

The article is organised as follows. Section 1 outlines one version of the enactive approach to cognition (sometimes referred to as ‘autopoietic enactivism’ (Hutto & Myin, 2013)).^7^ We focus here on this branch of enactivism in part because it provides a conceptual framework for understanding the strong continuity of lower and higher, or online and offline, cognition that is a central plank of our argument. We unpack the latter claim further in section 2 in explaining how proponents of the enactive theory have hitherto addressed the RHC. This leads us smoothly to section 3 in which we develop one of the central claims of our article that the enactivist’s response to the RHC can and should be recast in ecological terms. Section 3 shows how enactive cognitive science and ecological psychology might be combined and unified. Section 4 engages with some objections against such a proposal, from enactivists on one side and from ecological psychologists on the other. Finally, in section 5 we show how the result of our ecological-enactive synthesis of approaches can be put to work to reconceive representation-hungry cognition in ecological-enactive terms.

## 2. The enactive theory of cognition

The enactive theory of cognition takes as its starting point a theory of the self-organisation of living system as far from equilibrium, thermodynamically open systems that are able to produce and sustain their own organisation in their interactions with the environment. The organising principles that it takes to define what it is to be alive are then put to work to provide a theory of the cognitive.

Living systems, as it is argued, owe their continued existence to the property of organisational (or what is sometimes also referred to as ‘operational’) closure.^8^ A system that is organisationally closed is composed of dynamical processes [*P*_1_, *P*_2_, . . ., *P*_n_] such that ‘(1) the processes are related as network, so that they recursively depend on each other in the generation and realisation of the processes themselves, and (2) they constitute the system as a unity recognisable in the space (domain) in which the processes exist’ (Varela, 1979, p. 55).

To say that each of the processes [*P*_1_, *P*_2_, . . ., *P*_n_] ‘recursively depends on the others’ is to claim that each process is both a causal enabling condition and simultaneously an effect of the other processes that make up the system *S*. Each process exists for the sake (i.e. is a means for the production) of the other processes and is also a product (i.e. an end) of the activity of the other processes. The component processes of a living system thus stand in mutually productive relations. The system holds together over time only based on the activity of the processes of which it is composed. Each component process functions as an enabling condition for the production of the other processes that make up the system. Recursive dependence of this kind between processes yields an organisational property often referred to as ‘autopoiesis’ or ‘self-production’ (Maturana & Varela, 1980; Varela, 1979).

‘Autopoiesis’ refers to the process of continually regenerating the organisation of a living system through energetic and material exchanges with the environment, and their internal transformation and metabolisation. Autopoiesis has as a consequence that the system distinguishes itself from its environment. We observe this phenomenon at the most basic biological level of the living cell.^9^ The autocatalytic reactions that take place within the interior of the cell are enabled in part by the cell membrane that ensures that the catalysts are enclosed and do not defuse out into the environment. On the other hand, the membrane is generated and regenerated by the metabolic reactions taking place inside of the cell, in part based on the nutrients entering the cell from the outside. In this way, the cell creates a boundary around itself that distinguishes the cell from its surrounding environment (Di Paolo & Thompson, 2014, p. 90). We will refer to this property as ‘self-distinction’.

Any organisationally closed system will also exhibit the property of precariousness (Di Paolo & Thompson, 2014, p. 91). The processes that make up a living system are mutually productive such that if any one of them breaks down, this can have catastrophic consequences for the rest of the system. Each constituent process therefore depends on the others for its continued functioning. It will stop working without the activity of the other processes. What sustains the process and keeps it going over time are the joint activities of the other constituent processes of the network. The individual constituent processes have a natural tendency to decay if left to their own devices. This natural tendency towards ‘internal imbalance’ is corrected for by the activity of the other processes (Di Paolo & Thompson, 2014, p. 91). The result of the activity of these processes is a global organisation that is sustained over time in spite of tendencies of the individual processes to stop working, a possibility that presents a threat to the system’s ongoing viability.

Systems with this dual profile of organisational closure and precariousness are called ‘autonomous’ systems. They are described as *autonomous* first of all because their activities are based on ‘norms’ of the system’s own making. We can understand talk of ‘norms’ here as referring to global constraints on the system’s behaviour that arise from the system’s autonomous organisation. An example would be the metabolic need of an organism to keep its blood sugar levels within a certain range of values. The living system regulates its interactions with the environment in accordance with these global constraints. Interactions with the environment can be good or bad, adequate or inadequate, better or worse, correct or incorrect depending upon how they bear on the system’s concern to maintain its own organisational viability. What is good or bad for a living system is therefore defined (at least in part) in relation to the system’s organisational dynamics.^10^

Organisational closure makes it the case that the living system despite its precariousness is able to remain a self-sustaining unity over time. It gathers the energy and matter it needs, varying its relations to the environment in such a way as to resist an intrinsic tendency to disorder. In producing and sustaining its own identity under these precarious conditions, it establishes its own lived perspective on the world. From this perspective, the living system can register whether changes to its own internal conditions induced by the environment result in an improvement or deterioration in its conditions. It can register the extent to which it is deviating from ‘its optimal conditions of activity and its proper manner of realising equilibrium’ with the environment (quote is from Merleau-Ponty, 1963, p. 148). Lifeforms capable of movement can adaptively regulate their coupling with the environment so as to maintain their identity.

The individual’s meaningful or intentional relation to the environment is therefore established on the basis of its autonomy. The autonomous system acts with the aim of sustaining its own existence. Exchanges with the world are meaningful insofar as they affect either positively or negatively the viability of a self-sustaining and precarious network of processes (Di Paolo et al., 2010, p. 48). The source of the individual’s meaningful relation to the environment is deviations and departures from its optimal relation to the environment. Such deviations present a threat to its ongoing integrity, which the individual stands ready to take action to correct. Di Paolo and colleagues (2010) offer the example of a drug that a patient takes to treat a life-threatening illness. The drug is poisonous to the body so the patient has to slowly increase their dosage. Gradually, over time, the patient’s body adjusts to metabolising larger doses of the drug that would prove poisonous to the average person. The drug however does not have the negative value of a poison for the patient; on the contrary, the drug has positive metabolic relevance for the patient since the viability of their existence as a self-sustaining unity has come to depend upon it.

Living systems as autonomous systems stand in contrast to heteronomous systems that lack organisational closure and are therefore unable to build and rebuild themselves to avoid breaking down. An example of a simple heteronomous system is a thermostat that can be programmed to regulate room temperature. Heteronomous systems do not actively produce and maintain their own organisation under precarious conditions. This lack of organisational closure has the consequence that heteronomous systems have no ‘purposes’ of their own. The purposes or goals of the system come from its design and are thus external to the explanation of the workings of its component parts and their organisation. A heteronomous system can of course react to environmental conditions, and it can make use of sensory feedback to regulate its behaviour. It can also produce behaviour based on internal commands. What it does not do is act based on norms and values of its own making. It cannot set up a meaningful relation to the world on the basis of its own self-producing and self-maintaining organisation since it lacks organisational closure.

Computational systems, the behaviour of which is the outcome of building, storing and manipulating internal representations, are also heteronomous systems. It is because of their heteronomy that they must guide their behaviour in the world based on internal representations and goals that come from their programming much like the thermostat.^11^ Autonomous systems by contrast have no need for programmed goals and representations; they have purposes of their own that arise out of the struggle to sustain their identity through the regulation of their coupling with the environment. Based on the precarious conditions of its own existence, an autonomous system determines its own possible domain of interactions with the environment. It establishes its own meaningful or intentional relationship to the world in producing and sustaining its existence.

The enactive theory thus rejects representation in part because of its theoretical assumptions about the nature of cognitive systems as autonomous systems. An autonomous system does not relate to its environment through the mediation of representation as would be necessary for heteronomous systems that have no purposes of their own. The autonomous system as a self-producing, self-sustaining unity is selectively open to the milieu that it inhabits based on what matters to it, its living cares and concern. In the simplest of living creatures such as plants and single-celled bacteria, the environment consists of forms of matter and energy the living system needs to actively procure if it is to maintain the conditions of its own existence.

Thompson (2007) refers to this orientation to the environment based on the organism’s energetic and material needs as an orientation to the ‘virtual’ (p. 74). The term ‘virtual’ refers to ‘that which is real but not actualised’ (Di Paolo, Buhrmann, & Barandiaran, 2017, p. 228). The organism does not just regulate its interactions with the environment based on its sense of real and present conditions. It is also differentially sensitive to configurations of the agent–environment system that are only virtual. Thus, the status of sucrose as food-for-bacteria is real, but it only gets actualised when bacteria swim up the sucrose gradient (Di Paolo et al., 2017). It is only in relation to bacteria and their swimming activity that sucrose actualises its status as food. What is true of the environment of such simple creatures as bacteria is also true of more complex lifeforms. More complex agents are likewise selectively open and differentially sensitive to an environment of potentialities and possibilities based on norms that originate in their own autonomy, in the sustaining of their own identity as individuals. More generally, the significance the environment has for agents is virtual. This significance finds its actualisation – it is enacted – through the agent’s active engagement with the environment.

This enactive understanding of living systems and cognitive systems as sharing core organisational properties of organisational closure and precariousness thus presents a challenge to the computer theory of mind as the framework for cognitive science. Specifically, it calls into question the claim that the behaviour of cognitive systems in relation to their environment must be the outcome of computational operations carried out on information-bearing states. Computational systems as we have seen are heteronomous in terms of their organisational properties. They relate to their environments through the processing of what from an observer’s perspective can be characterised as information-bearing internal representations. Living systems by contrast are autonomous; they can regulate their interactions with the environment on the basis of their own precarious organisational closure. They have an individuality and identity, and based on this identity they are differentially sensitive to an environment of things that matter to them and are thus meaningful and valuable. Insofar as cognitive systems are understood as having autonomy, they cannot be computational systems, even though it may sometimes be explanatorily useful to treat them as such.

Can such a reconceptualisation of cognition in terms of autonomy explain cognition as it is put to work in representation-hungry domains? One might think not. By taking the environment to consist of virtual conditions that the living being enacts through its activities, the enactive theory seems to bind organism and environment together too tightly for it to explain cognition in representation-hungry cases. The RHC arises for cognitive processes that seem to be best characterised in terms of decoupled processes of reasoning in which the thinker is in some sense separated and detached from the object of its thinking because the object is abstract or absent (Clark & Toribio, 1994). The enactive answer to the RHC will require further clarifying what it means to think of the agent–environment system in terms of virtual conditions. It is here that ecological psychology can help, as we set about explaining in section 3.

## 3. From biology to cognition: agents as bundles of micro-identities

So far, we have explained something of how enactivists have characterised life in terms of autonomy. We have seen how the concept of autonomy provides the foundations for the enactive theory of cognition. However, as Barandiaran and Moreno (2006) have argued, it would be a mistake to simply identify cognition with the autonomy of living systems.^12^ The normativity that governs cognitive systems is not straightforwardly equivalent to that which governs life, as can be seen by reflecting on the breakdown of cognitive functions:

Failure to satisfy a cognitive purpose does not *necessarily* imply failure of material self-maintenance. The opposite of cognition and cognitive success is not death or biological illness but. . .some kind of coma, ‘madness’ or loss of behavioural coherence. (p. 175)

Failures of biological autonomy such as suicide can likewise involve significant exercise of a person’s cognitive resources in the form of planning for instance. Moreover, many cognitive skills such as chess involve responsiveness to norms that have little if anything to do with biological autonomy.

The solution to this problem lies in recognising that each living system produces and sustains multiple identities over the course of its life. Cognition is not best understood at the basic level of biological autonomy associated with metabolism (though metabolism will certainly play a central role in cognition through the dense reciprocal interactions between bodily affective processes and cognitive processes (Colombetti, 2014; Pessoa, 2013)). We should instead look for an explanation of cognition at higher levels of autonomy (Barandiaran, 2017; Barandiaran & Moreno, 2006).

Recognising that autonomy operates at multiple, interacting levels of organisation also serves as the starting point for the enactive theory’s response to the RHC. As the organisation of the autonomous system becomes more complex and its actions less bound by its immediate metabolic needs, the possible meaningful relations the organism can stand in to the environment become less tightly bound to the here and now. As its organisation grows in complexity, the virtual conditions to which the organism is sensitive increase in their temporal reach. Following Di Paolo et al. (2017), we suggest interpreting virtual conditions as the set of states of the agent–environment system that ‘surround a currently actualised trajectory of states’ (pp. 228–229). We take the ‘currently actualised trajectory of states’ to refer to the evolution of the variables that describe the changes in the states of the agent–environment system. These states could be plotted as a curve or trajectory through a multi-dimensional state space that describes the possible states of the agent–environment system. The states that surround the currently actualised trajectory are thus the locations in the state space the agent could potentially come to visit through its own activities. The agent is sensitive to the tendencies and trajectories that constitute the dynamical configurations of the agent–environment system, and the consequences of these tendencies and trajectories for its precarious existence. As the agent grows in complexity, it becomes sensitive to tendencies and trajectories in the evolution of its own states in relation to the environment that stretch steadily further through time.

We see the beginnings of this temporally extended engagement with the virtual already in the simplest of creatures. Bacteria such as *E. coli* have a minimal form of agency (Di Paolo et al., 2017).^13^ They are differentially sensitive to the conditions of their environment based on their metabolic needs, and switch between behaviours according to what their metabolism demands of them. Minimal agents regulate their coupling to world based on metabolic relevance. The chemotactic behaviour of the single-celled bacterium as it tumbles about in search of sucrose exhibits a minimal degree of selective openness to its environment. The bacterium does not find itself as if by magic in the presence of a sucrose gradient. It has to first randomly search for locations in its environment that offer a source of nutrition. Once it hits upon such a location, it actively orients itself towards the greatest concentrations of nutrition by gradient following. Similarly, any creature that is capable of avoidance behaviour (as observed in bacteria when they move away from potentially noxious substances such as alcohols and fatty acids) can relate to possible dangers that lie ahead, but are not immediately present at its current location. Such a creature is already exhibiting a minimal degree of prospection, dealing with the virtual (real but not yet actualised) possibilities that lie beyond the reach of the here and now.

As far as we know, bacteria do not enact values that go beyond what is metabolically relevant to them. However, in creatures with more complex biological organisations, patterns of sensorimotor behaviour can quite literally take on a life of their own (Di Paolo et al., 2017; Egbert & Barandiaran, 2014). In the biological world, we find networks of processes that produce and sustain an identity at multiple levels of organisation from ‘microbial communities, nervous systems, immune systems, multicellular organisms, ecosystems, and so on’ (Thompson, 2007, p. 46; c.f. Varela, 1991, 1997).^14^ We interpret the enactivist’s concept of ‘identity’ to refer to the biological organisation of an individual that is maintained over time through material and energetic exchanges with the environment. Varela (1991, 1997, 1999) proposed thinking of agents as bundles of ‘micro-identities’. The immune system produces and sustains a coherent somatic identity at the molecular and cellular levels of organisation by meeting the energetic needs of individual cells. The nervous system generates and maintains stable and recurrent patterns of sensorimotor engagement with the world. It couples movement to sensory activity, linking the sense organs and their nerve endings to effectors in the body in cycles of perception and action. Perception and action are codetermining: what the animal senses depends on how it moves, and its movement depends in turn on what it senses (Thompson, 2007, p. 47; c.f. Gibson, 1966, 1979). The network of processes in the nervous system that link sensory activity to movement ‘closes back on itself’ (Thompson, 2007, p. 50): any change in activity in sensory neurons leads to change in motor neurons, and vice versa.

Agents are in this sense made up of bundles of interacting micro-identities (Varela, 1999). Micro-identities combine to form an agent’s perspective on its environment relative to which things are encountered as being valuable or meaningful. Varela (1999) describes each micro-identity as a ‘readiness-for-action proper to. . . a specific lived situation’ (p. 9). The cycles of perception and action the nervous system holds together in an organisationally closed network are recurrent patterns of practical engagement with familiar everyday situations. Varela suggested that we think of each state of action readiness as a self-sustaining, self-producing micro-identity that gets enacted as an individual takes part in a regular, everyday activity. As the individual aims for the continuity of each of its micro-identities in its coupling with the environment, it ‘brings forth’ or ‘enacts’ what Varela described as a ‘microworld’ – a recurrent pattern of interaction with the environment. Varela gives the example of sitting down to eat dinner with the family as an example of such a recurrent situation. As we prepare to take a seat around the table, we are ready to perform multiple actions: we are ready to sit in the chairs that are placed around the table, take hold of the cutlery we use to eat, make conversation with the other members of the family present for dinner and so on.

The growth in the complexity of agency can thus be understood in terms of the complexity in the patterns of an agent’s behaviour – the recurrent patterns of interaction with the environment the agent sustains over time. We started this section by arguing that as the agent grows in complexity the virtual conditions to which it is sensitive will also increasingly extend their reach through space and time. This point is crucial to understand how higher cognition could be strictly continuous with lower cognition. Higher cognition is characterised by abilities to deal with the distal, the possible and the abstract. We have begun to develop an analysis of such abilities in terms of the spatial and temporal reach of sensitivity to virtual conditions. To complete this argument will however require adequately accounting for what Varela calls the ‘lived situation’ to which every readiness-to-act is correlated. As agents develop in complexity so also does the environment they are able to deal with. In the next section, we will show how doing full justice to this point should lead enactivists to join forces with ecological psychologists.

## 4. Situating enactive cognition in a landscape of affordances

While we have followed Varela in suggesting that agents be thought of as bundles of micro-identities, this should not be taken to imply that the individual is a simple aggregate of its micro-identities (Bruineberg & Rietveld, 2014; Di Paolo et al., 2017, ch. 6). Instead, micro-identities are interrelated in such a way as to form interrelated patterns of sensorimotor engagement with the environment. Thus, the activity of eating at the family table is connected to habits of what the family typically eats that have been established in the past, the activities of preparing food, shopping, working, social relations within the family unit and so on.

The same is also true of what Varela (1999) called ‘micro-worlds’. To recognise this point requires however paying close attention to the ways in which the environment is both structured by, and also always already structuring, our activities. Each micro-world has its own complex internal structure and enters into a set of tangled relations with other micro-worlds. A working day, for instance, consists of multiple, interrelated activities, each exhibiting its own nested structure integrated to form a meaningful whole. Each activity depends for its self-maintenance on the mutually enabling interrelations it forms with other activities. Environmental structures are also a part of what gets sustained over time. One’s working day consists of well-worn grooves in part because of the behaviour settings in which we do our work (Barker, 1968; Heft, 2001 chs 7 & 8; McGann, 2014).^15^ The term ‘behaviour setting’ is borrowed from the ecological psychologist Roger Barker. It refers to the regular and stable patterns of interaction among multiple individuals that constrain what people do as they act in a particular place such as an office, a classroom, a drugstore or a public park. When we enter an office, we ready ourselves for certain possibilities – the colleagues we are likely to meet, the activities we regularly perform while at work, the interaction with our boss, the route we need to take to reach our office and so on. There are constraints in play in this setting because of the regular and stable patterns of activity that have established themselves over time as people act and interact in this place. In a public park, regular and relatively stable patterns of social interaction play out in part because of structures that are placed around the park. People may take a seat on a park bench taking care not to sit too close to the stranger already seated. Other people run through the park along paths already in place, taking care not to collide with the people out for a walk with their dogs. On a hot summer’s day, visitors may take a swim in the lake, assuming the rules of the park allow for swimming.

Thus, we should not think of the organisational closure at the level of an individual’s activities just as the product of recurrent patterns of neural activity taken in isolation from the rest of the body and the settings in which our activities regularly take place. Instead, we should think of these patterns of neural activity as depending upon agent–environment couplings (Bruineberg & Rietveld, 2014; c.f. Di Paolo et al., 2017, p. 152). Everyday activities form networks of self-sustaining processes that have among their component parts neural, bodily and environmental elements.

Elsewhere, we have described this phenomenon of the agent’s coordinated interaction with a web of interrelated situations in terms of responsiveness to a landscape of affordances (Rietveld & Kiverstein, 2014). The aspects of the environment such as the chair, the dining table, the cutlery and so on all provide possibilities for action. In other words, they offer affordances, which we define as relations between aspects of a (socio-material) environment and the skills and abilities available in a form of life. The term ‘form of life’ (Wittgenstein, 1953) refers to the regular ways of doing things and steady ways of living that can be observed in groups of animals. One and the same affordance is typically caught up in a variety of very different activities (Bruineberg & Rietveld, 2014; Van Dijk & Rietveld, 2016). Thus, chairs offer the possibility to sit, but sitting is itself an activity that can be performed in a variety of different contexts and settings from dining to working, socialising with friends and so on. Moreover, this is clearly not all that one can do with chairs: they can, for instance, be used for firewood, in a children’s party game, and even play the role of an example in a philosophical argument. Affordances occupy a place in historically developed constellations of regular, more or less stable patterns of activities that make up a form of life.^16^ We talk of ‘responsiveness’ to affordances to highlight how affordances constrain what people do. The individual’s engagement with affordances is skilful; their performance is subject to normative assessment as better or worse, as more or less correct given the specific demands of the situation (Rietveld & Kiverstein, 2014, p. 332).

We talk of a ‘landscape of affordances’ to capture the richness and interrelatedness of the affordances the environment offers. Landscapes have a complex nested structure as Gibson (1979) notes: ‘canyons are nested within mountains; trees are nested within canyons; leaves are nested within trees; cells are nested within leaves. There are forms within forms both up and down the scale of size’ (p. 5). Affordances likewise exhibit these relations of nesting at multiple spatial and temporal scales. The relations between affordances in the landscape of affordances are a reflection of the interrelated activities engaged in by members of a form of life, the patterns in their behaviour. Thus, when I enter a library, for instance, the first thing I might do is switch my phone to silent in preparation for the silent reading areas I am about to enter. The affordances of the library as a place constrain my behaviour over a relatively long timescale (Bruineberg & Rietveld, 2014). The affordances of objects in the library such as the door handle I use to enter one of the rooms in the library constrain my behaviour over shorter timescales. As soon as I enter the library building, my body is already preparing for action over these multiple timescales. My patterned states of action readiness are coordinated to (i.e. constrained by) the nested affordances of this place.

We suggest then that micro-identities are best understood in ecological-enactive terms as interrelated states of action-readiness that coordinate to multiple relevant affordances. Agents are made up of a multitude of interrelated micro-identities. They are not bound to the here and now because they are always selectively open to multiple possibilities simultaneously at multiple scales (Bruineberg, Kiverstein, & Rietveld, 2016; Bruineberg & Rietveld, 2014).

The ‘virtual’ conditions to which the individual is differentially sensitive should, we suggest, be thought of as a landscape of affordances. Organisms in general we have seen act in the here and now, but with ‘reference to a spread-out environment…an environment that is both extensive and enduring’ (Di Paolo et al., 2017, p. 230; quotes are taken from Dewey, 1929, p. 279). The human environment in which people act has been structured over long periods of times by our social and cultural activities. Individuals develop in these socio-material practices and acquire sensitivity to the proper ways of taking part in those practices (Rietveld, 2008; Rietveld & Kiverstein, 2014). Based on their history of taking part in practices, they are able to selectively engage with relevant affordances over longer temporal scales, ‘projecting’ their sensitivity to the possibilities the environment offers further and further into the future. For example, an expert ice climber is not just preparing herself for the next move but for the whole trajectory ahead. She is responsive to the multiplicity of affordances as a whole, ready to make a move that will put her on the right track for the whole route ahead (Bruineberg & Rietveld, 2014; Seifert et al., 2014).

The role of affordances in enaction has only occasionally been recognised in the enactivist literature, and when it has enactivists have had relatively little to say on the matter.^17^ Indeed, Varela et al. (1991) were critical of ecological psychology for claiming that an animal’s niche ‘does not depend in any way on the perceptually guided activity of the animal’ (p. 203). They contrast what they take to be Gibson’s one-sided view of the animal–environment with their own view of the animal–environment relation as ‘the enactment or bringing forth of a world by a history of structural coupling’ (p. 205).^18^ On the other hand, in recent years ecological psychologists have expressed some scepticism about whether an enactive theory of cognition is even needed (Fultot, Nie, & Carello, 2016). They have noted some points of tension with ecological psychology. By contrast, we have been arguing that ecological psychology and enactive cognitive science form natural partners and developed a rich notion of skilled engagement with a rich landscape of affordances that can do justice to the insights of both sides. In the next section, we defend the possibility of such a joint ecological and enactive research programme against sceptics from both camps that might query the need for such a programme.

## 5. Towards an ecological-enactive approach to cognitive science

In early treatments of autonomy, organisational closure was given an interpretation in such a way as to render the role of the environment in autonomy seemingly irrelevant. Thus, Varela (1979) wrote: ‘the environmental elements intervening between the effector and the sensory surface of the organism is irrelevant, because the nervous system can be refined as a network of neuronal interactions in terms of the interactions of its component neurons, regardless of intervening elements’ (p. 242). Consider in this light Maturana and Varela’s (1987) well-known claim that the brain can be compared to the navigator of a submarine that has epistemic access only to the ‘indicator readings, their transitions, and ways of obtaining specific relations between them’ (p. 137). The environment is cast in the role of, at best, perturbing the internal dynamics of the nervous system. Intervening environmental elements are irrelevant because it is the activity internal to the nervous system that determines how the organism responds to environmental perturbations. Maturana and Varela (1980) insisted on a distinction between what they called the ‘relational’ and ‘operational’ domains. The operational domain describes the organisationally closed network of processes that make up an autonomous system and the way in which those processes close in on themselves. In the operational domain, the environment is only the source of perturbations. It is only by taking up the standpoint of an observer that we can gain epistemic access to the relational domain: the domain that forms through the coupling between organisationally closed networks of processes and their environment.^19^

Enaction is often described as the ‘bringing-forth of a world’, terminology that has idealistic connotations of the agent somehow bringing the world into existence through their activities. These idealistic themes are strongly compounded by the early work on autonomy just mentioned in which the environment is cast as relevant to autonomy only from an observer’s perspective. It is however hard to square internalism of this type with frequently encountered passages in the more recent writings of enactivists in which organism and environment are described in terms of ‘co-determination’ and ‘mutual interdependence’ (Varela et al., 1991, p. 177; also see Di Paolo, 2018; McGann, 2014; Thompson, 2007, pp. 152–154). A strictly internalist perspective on autonomy also seems to be in tension with a view of the individual organism as made up of interrelated micro-identities. We argued above that each micro-identity as a state of action-readiness is best understood in relation to a relevant affordance. Moreover, states of action-readiness do not occur in isolation; they stand in complex patterns of interrelatedness that allow the individual to coordinate their behaviour to multiple relevant affordances at the same time.

The enactivist’s talk of ‘sense-making’ may evoke ideas of the organism generating meaning from inside of itself, and projecting this meaning onto an otherwise meaningless physical world. We have suggested however that sense-making is best understood as referring to the affective significance the environment has for an individual agent. Sense-making is thus best understood in relational terms as arising in the agent’s coupling with relevant affordances in its environment. The individual agent has a perspective on its environment relative to which it encounters affordances as presenting an opportunity or threat to the sustaining of its micro-identities. Some affordances have a positive valence drawing the agent into action. Others have a negative valence repelling the agent away from them. Affordances have a positive or negative valence due to the organism’s sense-making capacities. ‘Sense-making’, as we understand this notion, should be understood in relation to the agent’s skills and abilities that make them ready to respond with varying degrees of urgency to multiple relevant affordances.

Elsewhere, we have characterised these agentive capacities in providing a more general account of *skilled intentionality* as the tendency towards an optimal grip on multiple affordances simultaneously (Bruineberg & Rietveld, 2014; Rietveld, Denys, & Van Westen, in press).^20^ A lack of grip on the environment can manifest for the subject as an experienced tension. An example is the tension one experiences when someone stands too close to us in conversation (Dreyfus & Kelly, 2007; Rietveld, 2008). The agent might then be drawn into taking a step back so as to reduce the tension. On our account, it is on the basis of the tendency towards an optimal grip that affordances have the valence they do, some standing out as attractive, others as repulsive, still others not moving us at all. Those affordances that ready the agent for action are possibilities for action that contribute to improving its grip, moving the organism closer towards being in a state of (relative and unstable) equilibrium with the environment.

The notion of affective significance we have appealed to in explaining the enactive notion of sense-making *presupposes* the meaning that is already present in the environment because of the affordances it offers (Hodges & Baron, 1992). This dependence of sense-making on the affordances of the environment can be seen in the following two ways. First, we have defined affordances in relation to forms of life. Sense-making as we have characterised it applies to *relevant* affordances for a particular individual – the possibilities for action the environment offers that bears either positively or negatively on an agent’s micro-identities. Sense-making thus presupposes skills and abilities on the part of the individual for coordinating their behaviour to relevant affordances. Such skills and abilities sensitively attune the individual to the meaning that is present in the environment because of its affordances.

Second, many of the affordances of the human environment grow out of people’s participation in socio-material practices. What they come to care about is to a large extent a reflection of their being a member of an interrelated web of practices. When they relate to certain affordances as bearing positively or negatively on them, this is often because those affordances relate in some way to the wider practices in which they participate. It is on the basis of this participation in practices that the individual has a sense of what is appropriate and inappropriate and thus of how to go on correctly in a given situation (Rietveld, 2008; Rietveld & Kiverstein, 2014). In other words, the person’s lived and situated normativity is sensitive to a normativity found at the level of the forms of life to which they belong. When certain affordances stand out as significant drawing the agent into action, they do so because the individual is sensitive to what counts as going on adequately in the practice. For example, I drink from my own glass and not from your glass when we dine together.

In the ecological psychology literature, affordances have often been taken to be properties of the environment that can be directly perceived because perceptual systems are attuned to a structured ambient array that carries information about affordances (see, e.g., Fultot et al., 2016; Turvey, Shaw, Reed, & Mace, 1981). The ambient array carries information about affordances by being patterned and structured in ways that stand in law-like relations of correspondence with the layout of the environment. This structure in the ambient array makes it possible for the perceptual systems that are attuned to it to pick up information that tells the perceiving animal what it can do in its local environment.

Our relational account of affordances does not tie an animal’s sensitivity to affordances tightly to information contained in the ambient array that stands in law-like relations to the animal’s surrounding environment. Indeed, we would argue that the ambient array does not contain information about affordances independently of how the structure in the ambient array can be used to do things by the animals *in the form of life* (Hutto & Myin, 2017; Van Dijk, Withagen, & Bongers, 2015). Instead, we understand direct perception as the skilled activities that animals perform in sustaining coordination to affordances. In the case of people, many of these activities are performed by taking part in patterns of practice and by coordinating to the affordances of the environment in ways laid out in such practices. People enter situations ready to respond to their affordances based on their skills. These are skills people find themselves with because of their familiarity with ongoing socio-material practices.

The enactive theory of sense-making thus needs to be combined with the ecological theory as we have developed it, if it is to account for the meaning and normativity already in place in the collective patterns of activities that structure the human landscape of affordances. It is indeed necessary to talk about the sense-making activity of the individual agent. We characterise this sense-making activity in terms of the tendency towards an optimal grip on multiple affordances (i.e. in terms of skilled intentionality). However, we argue that often affordances show up for an individual as affectively significant only because of their wider involvement in regular and stable patterns of activity sustained over time in the socio-cultural practices to which they belong.

To summarise, we have proposed the following synthesis of ecological and enactive theories. Enactive theories are needed to understand the strict continuity of lower and higher cognition. Here we have made appeal to recent work on living systems as exhibiting organisational closure across multiple scales of complexity (Di Paolo et al., 2017). In addition, enactive theories provide a complementary conceptual framework for understanding an individual’s selective openness to affordances to the one we have elsewhere provided (Bruineberg & Rietveld, 2014).

Ecological psychology provides a conceptual framework for understanding how an individual can coordinate their activities to the affordances available in the surrounding environment and the wider social and material practices in which those affordances take shape. Such a conceptual framework is necessary for understanding the concept of the ‘virtual’ employed to characterise the individual’s responsiveness to possibilities that are real but not yet actualised. We saw above how as agents grew in complexity they were able to extend the reach of their sensitivity to the virtual through space and time. Ecological psychology is necessary for explaining this sensitivity to the virtual in terms of interrelated abilities for navigating a rich landscape of affordances. We return now to the task of developing an ecological-enactive account of representation-hungry cognition.

## 6. Representation-hungry cognition reconceived

The RHC was originally framed by Clark and Toribio (1994) around two classes of cognitive processes. In the first class of representation-hungry cases, agents think about something absent from their environment. The absent entity could be distally present, non-existent or counterfactual. Thus, in imagination, memory and planning for instance a thinker engages in thought processes that are directed towards entities that are not currently present in the environment and might never have been. The second class of representation-hungry cognitive processes involves abstract thought of some form. What this class of thoughts shares in common is that they involve thinking about some property at a level of generality that renders the property in question unobservable. We find abstract thought at work in categorisation to give just one example. People are able to place items into different categories often based on exposure to just a few items that may share little in common in terms of their observable properties. They can do so by identifying patterns of similarity despite, as Clark and Toribio (1994) put it, the ‘ambient physical manifestations’ of those properties being ‘complex and unruly’ (p. 419). These two classes of representation-hungry cognitive processes seem to share in common the property of *being decoupled from the environment*. Thus, in imagination and memory one seems to be not currently in contact with or coupled to the object of one’s imagination or memory. This can lie somewhere in the distant past in the case of memory, and for imagination in the possible future that is yet to occur, and may never happen. In abstract thought, one engages in thinking about some property one is not currently in contact with because the similarity one’s thought tracks goes beyond any concretely observable property of the type one could couple to.

In the presentation of our ecological-enactive theory of cognition, we have been putting pressure on the characterisation of so-called representation-hungry cases of cognition in terms of decoupled reasoning. Skilled intentionality, the coordination with multiple affordances simultaneously, is central in our ecological-enactive approach to ‘higher’ cognition. We have shown how enaction is best understood in an ecological context of nested agent–environment relations, spanning multiple scales of complexity, many of which reach far beyond what is taking place here and now. I can, for instance, use the affordances of my watch to ensure that my activities over the course of the afternoon are coordinated to the 17.30 train that will leave the train station located in the city centre in time to take me home for dinner when my family are expecting me (Bruineberg, Chemero, & Rietveld, 2018). The activity of looking at my watch and the affordances it offers me for telling the time are interrelated with a host of other activities such as leaving my office at a certain time, locking the door, taking my normal route to the station, dealing with the transport system and so on. The activities I perform over this longer timescale place constraints on what I do over shorter timescales. I am dealing with the absent – in this example, the train that will depart later in the day from a location on the other side of town – by coordinating multiple nested activities to the relevant affordances of the environment.

We should not think of this process of coordination in terms of decoupled reasoning because the process of coordination is not instantiated independently from what is happening in the environment. Each of the interrelated activities is coupled to the environment insofar as it takes the form of an affordance-related state of action-readiness. The agent is continuously, through this whole temporally extended process, preparing to take actions that it anticipates will improve its grip on the temporally unfolding situation. In this process, they adapt their state of preparedness to unexpected events as they arise in a dynamically changing environment.

There is thus no basis for positing a sharp conceptual divide between online and offline cognition. Online control is typically distinguished from offline cognition on the basis of decoupling (Clark & Grush, 1999). We have shown how a strict continuity of online and offline cognition might be defended in which offline cognition is instead viewed as a complexification and elaboration that grows out of online cognition. As cognitive agents grew in complexity, they were able to engage in activities that were coordinated to progressively more complex nested structures in the environment that span increasingly long stretches of time.^21^ We should not think of offline cognition as a distinct type of cognition, but as a more complex form of coordinating nested states of action readiness and activities to multiple relevant affordances. Such a process is complex because of the nesting of the activities and their increasing reach through time.

Still one might wonder whether we have really accomplished what we advertised: have we really provided a reconceptualisation of representation-hungry cognition? So far, all we have done is provide a framework for thinking about how representation-hungry cognition could be accomplished without decoupled reasoning. This does not yet amount to providing the concrete details of how an agent could engage in imagining, remembering and abstract thinking without making use of internal representations. Consider the following case from Shapiro (2013). Shapiro offers a description of himself deliberately imagining revisiting his house in New Jersey where he grew up in the 1970s. He describes the layout of the house; the location of the bathroom; the decorations in his bedroom; the Jimi Hendrix poster hanging on his bedroom wall. None of these things are present to Shapiro here and now. We can suppose he has not even given them any thought for decades. Yet he is able to intentionally conjure them up in his imagination now and convey something of what he is imagining to us as his readers through the medium of written language. How can Shapiro do any of this without making use of internal mental representations?

The type of imagining involved in Shapiro’s example involves a kind of visualisation in which Shapiro deliberately re-enacts past experiences by remembering. We will call this ‘episodic memory (EM-) imagining’ following Langland-Hassan (2015). Shapiro reactivates some past experience thereby bringing into the present in his episode of imagining something from his past. It is surely tempting to conclude that the only way he can do this is by having some internal state that has the functional role of standing in for the features of the home from his childhood in their absence. At the beginning of our article, we have defined representations as states of a system that function as stand-ins or surrogates for things or states of affairs in their absence. So have not we just argued that the only way Shapiro can engage in this kind of elaborate visualisation based on memory processes is by making use of internal representational states?

How we answer this question will depend on whether imaginings that involve mental imagery must also be taken to have representational content. It may seem as if we have already provided an answer to this question a priori, based on the definition of representation we have adopted. However, what is in question in our article is the possibility of a positive alternative, namely, *whether representation-hungry cognition might be achieved by other means* – by, for example, skilfully coordinating complexly structured nested activities to multiple affordances. Could an account of EM-imagining also be given in these terms?

Notice that what Shapiro does to visualise his childhood home is re-enact past perceptual experiences. The result of this re-enactment is that he comes to occupy states that resemble in some way (or perhaps fail to resemble if he misremembers) the experiences he had when he was a child. We suggest understanding re-enactment in terms of states of action readiness. We have characterised perception as nested activities of coordinating to multiple relevant affordances simultaneously. When Shapiro re-enacts his past experiences, this can be thought of in terms of him pretending to engage in activities of coordinating to multiple relevant affordances, without going through the motions of actually doing so. There is some precedent for this way of thinking about EM-imagining in Gibson when he writes,

. . .a perceptual system that has become sensitised to certain invariants (information) and can extract them from the stimulus flux can also operate without the constraint of the stimulus flux. (Gibson, 1979, p. 256, quoted by Van Dijk & Withagen, 2016, p. 23)

Here Gibson seems to suggest that a perceptual system that is attuned to the structures of its environment could use this skilful attunement in the absence of this structure in the environment. What perceptual systems do is attune to the external dynamics in the environment (e.g. the pattern and structure in the ambient array) that allows the agent to maintain coordination to a relevant affordance. In sense perception, a perceiver maintains coordination to affordances by continually tending towards optimal grip, thereby reducing the disattunement between internal and external dynamics (Bruineberg & Rietveld, 2014).^22^

In EM-imagining, the perceiver relies on the very same attunement to external dynamics that is used in action to coordinate to affordances. Her attunement thus affords her another possible action – she can pretend to perform the very same activities she would perform when perceiving, for instance, the poster in the bedroom but without actually perceiving it. What she imagines then is dependent both on her abilities and the affordance offered by the environment she would coordinate to in perception. Sense perception does not involve representational content but is instead understood by us as a skilled activity of engaging with multiple affordances simultaneously. But note now that if sense perception does not involve representational content, then nor does EM-imagining (Degenaar & Myin, 2014; Hutto & Myin, 2017). EM-imaginings as we have characterised them are just activities of pretending to do what is done in perception. Re-enactments of non-representational processes do not become internal representations just by virtue of being re-enactments.

One might object that EM-imagining on our account re-enacts activities of coordinating to affordances that are currently absent. The subject pretends to perform the same activities they would perform were they coupled to affordances. But to pretend to perform those activities it might be argued that they need to make use of an internal model of affordances that acts as a stand-in for the absent affordances, guiding the subject’s re-enactment in the absence of the real thing (Foglia & Grush, 2011). The objection continues that to re-enact engaging in activities of coordinating to affordances requires the mock performance of activities in which one actually coordinates to affordances, albeit not in the actual world but only as represented in internal model. Thus, a block of wood might be used to actually perform a rotation task in which the task is to tell whether two objects would match in shape when rotated by 90 degrees. To pretend to engage in this rotation task, one would need to carry out operations on an internal model of the block of wood (Foglia & Grush, 2011).

We dispute however that it is necessary to invoke an internal model of affordances that the subject somehow manipulates to understand how a subject could pretend to coordinate to affordances that are absent. Models carry information about what they represent based on systematic patterns of covariation that hold between the model and whatever it is modelling in the world. Instead of locating such systematic structure-preserving patterns of covariation in the head, we suggest looking for them instead in the relations of covariation that hold between the patterns of action-readiness and affordances available in the landscape. What the subject does in EM-imagining is *pretend to enact the states of action-readiness that would typically enable them to coordinate to affordances*. This is something the agent can do based on their being attuned to the external dynamics that agents typically use to sustain coordination to affordances. EM-imagining is thus a possibility for action available to the individual because of their skills for coordinating to affordances, attuning to the structure in the environment. One of the things the agent can do with their skills is to re-enact what one would perceive were one actively coordinating to affordances present in the environment. Performing this re-enactment does not require the manipulation of any internal models.

Finally, it might be objected that EM-imagining is just one form of representation-hungry cognition. Does our ecological-enactive account have anything to say about other varieties of representation-hungry cognition such as creative imagination or abstract thought? In what is already a long paper, we cannot attempt to provide a full account of representation-hungry cognition in all of its different guises. With the caveat that we plan to fill out the details of these brief comments in future work, we finish up by saying something very brief about these other cases.

How would we reconceive abstract thought within our ecological-enactive framework? Here what is crucial to keep in mind (at least for humans) is *development* of children in a linguistic environment that scaffolds thinking, including abstract thought. Many of the abstract, complex and unruly similarities that children can rapidly become attuned to are similarities that they become familiar with through linguistic interaction with adults. What language is enabling the child to do is to attend to unruly patterns of similarity and difference that form the basis for abstract thought. Such unruly patterns are in the end ways of going on in linguistic practice to which the child learning abstract patterns of thought is being trained to coordinate. The linguistic activities of other people are being used to explore and find structure and patterns that allow the child to coordinate to the affordances of the linguistically structured environment. The capacity for abstract thought grows out of a potent combination of agents willing to submit to strict training regimes in which they soak up culturally pre-established practices including the affordances that language offers for engaging in abstract and symbolic modes of cognition.^23^

We suggest thinking of creative imagination by analogy with the capacity to create new possibilities for action in play activities.^24^ We propose to think of the creative imagination in terms of exploratory activities. Situated in a concrete situation, creative imagination opens one up to hitherto neglected affordances (Van Dijk & Rietveld, under review; Hutchins, 2010). Agents engage in activities that allow them to explore for possible structure, pattern and regularity in the environment before arriving at stable structures that allow them to maintain the coordination of their activities to affordances (Reed, 1996; Van Dijk & Withagen, 2016). The result of finding these novel patterns and structures is the coordination of activities to novel affordances. Thus, what creative imagination does as an exploratory activity is allow individuals to gain access to neglected or novel affordances and thus to expand the horizon of the field of relevant affordances in which they are situated (Rietveld & Kiverstein, 2014).^25^

## 7. Conclusion

We have shown how the ecological and enactive approaches can be combined to reconceive cases of allegedly representation-hungry cognition in non-representational terms. Our argument has proceeded in two steps. First, we have argued for a synthesis of enactive and ecological research programmes. This has required us to resolve certain tensions between these research programmes that have arisen in part from an unfortunate neglect of the contribution of the environment to cognition in the enactive literature. We have corrected for this neglect by providing an analysis of enaction in terms of nested activities that coordinate to multiple relevant affordances simultaneously. In our ecological-enactive framework, sense-making can then be understood as the tendency towards an optimal grip on multiple affordances simultaneously (i.e. as a form of skilled intentionality). Second, we have argued that complex agents capable of dealing with the so-called representation hungry domains are always coordinating not only to the here and now, but also to the absent, the possible and the abstract.

Representation-hungry cognition has struck many researchers as a source of hard cases for dynamical approaches to cognition such as enactive cognitive science and ecological psychology. We have argued that they have appeared to be hard cases because representation-hungry cognition has been understood in terms of decoupled reasoning. We have sought to break this conceptual connection by arguing that even when agents are dealing with the absent, the possible and the abstract they are still coordinating to the rich landscape of affordances available in their ecological niche. We have thereby made a start at showing how one might satisfy hunger for representations through enacting skilled engagement with multiple affordances simultaneously.

## Notes

1. Clark and Toribio were responding to arguments of dynamical systems theorists against the necessity for representational explanations of cognition. Enactive cognitive science shares much in common with these dynamical approaches (see Thompson, 2007, chs 1 & 3 for further discussion). The main difference is that the enactive theory provides an account of what makes a dynamical system a cognitive system, something that is missing from the original formulations of dynamical theories that were the target of Clark and Toribio. See Barandiaran and Moreno (2006) for more discussion of this last point.

2.
Hutto and Myin (2013) eschew explanations of what they call ‘non-basic’ cognition in terms of content-bearing *internal* mental representations. They do however continue to make reference to *external* content-bearing states (paradigms of which are public language inscriptions and utterances) to account for non-basic forms of cognition. We will not take a stand on whether higher cognition might require explanation in terms of content-bearing *external* representations. Our target in this article is cognition in the so-called representation-hungry domains. When we say that there are no cases of representation-hungry cognition, we mean that cognition in the so-called representation-hungry domains can be explained without making appeal to internal mental representations. We leave it as open possibility to be investigated in future work that some of these processes might need to be explained in terms of external systems of representation.

3. See, for example, Wheeler (2005), and more recently Clowes and Mendonça (2016) for defences of such a distinction.

4. Recall we define ‘internal representations’ as states of a system that stand in and that function as surrogates for things in their absence. Of course it is open to philosophers to question our definition of representation as overly restrictive. This opponent may agree with us that living systems certainly did not start out using internal states that stand in for external states of affairs. They began with simpler sensory systems that merely detected external states of affairs. However, gradually over the course of evolutionary history, creatures developed increasingly complex capacities for controlling their behaviour and began to acquire internal functional profiles that increasingly resembled full blown representations as we have characterised them. We cannot fully engage with such a line of argument in this article, but we note it rests upon reintroducing the very distinction between higher and lower forms of cognition we will set out in this article to challenge.

5.
McGann (2014) is an important exception, and more recently Di Paolo, Buhrmann, and Barandiaran (2017).

6. The social and the material are inextricably intertwined in such practices, and for this reason we will henceforth talk of socio-material practices (Van Dijk & Rietveld, 2016).

7. Hutto and Myin distinguish autopoietic enactivism (AE) from sensorimotor and radical versions of enactivism. We focus on AE here because it provides the overarching theoretical framework for the other forms of enactivism (see, e.g., Noë, 2004, ch. 7). Degenaar and O’Regan (2017) distinguish sensorimotor enactivism from AE enactivism. Engaging with their argument is however outside of the scope of this article.

8. Di Paolo and colleagues explain that the term ‘closure’ has its origins in mathematics. The mathematical concept of closure applies to sets that are closed under an operation in the sense that application of an operation to the elements of the set only produces other elements of the set. They give, as an example of such a set, the integers for the operations of addition and subtraction (Di Paolo et al., 2017, p. 112). The terms ‘organisational’ and ‘operational closure’ are used interchangeably in the literature. ‘Operational’ closure is used to denote that closure is ‘achieved through the actual work and transformations done by processes in time (i.e. we are not talking about closure in a formal, mathematical sense)’ (Di Paolo et al., 2017).

9. As we will discuss in more detail shortly, the same organisational properties can also be found at higher levels of organisational complexity.

10. It should be noted that this characterisation of the normativity that arises with autonomy is rather crude and does not capture the nuances in the evaluative relations autonomous systems can take up to the world. Di Paolo et al. (2017) describe living systems as being sensitive to the ‘risk of disintegration, or to *gradients* and *directions* in their viability conditions’ (p. 122). They use the term ‘adaptivity’ to refer to self-regulation based on more subtle changes in normative conditions.

11. Nowadays, some computers are capable of programming themselves: think of neural networks that are capable of unsupervised learning such as Deep Mind’s AlphaGo Zero that recently learned to play the ancient Chinese game of Go without human instruction, or machines that can improve each other’s software. We leave as an open question whether such machines are counter example to the argument we have given above. They seem to lack organisational closure and thus cannot set up a meaningful relation to an environment based on values grounded in the maintenance of this closure. They can only relate to the world by finding statistical patterns in the data they receive. Of course such machines excel in mining data for statistical patterns, but biological agents do not care about abstract statistical patterns in themselves but only insofar as they bear on their practical concerns. Finding abstract statistical structure does not suffice for establishing a meaningful relationship with the environment; this requires in addition the concerns of a living creature.

12. See also Di Paolo (2005).

13. Although we describe *E. Coli* as examples of ‘minimal agency’, we do not mean to imply that the behaviour of *E. Coli* is simple and mechanical. Nor we take it do Di Paolo and colleagues (2017) from whom we borrow this term. We focus on chemotaxis in our brief discussion above, but as Alexandre (2010) discusses bacteria make use of Energy Taxis receptors that allow cells to flexibly couple motility behaviours with their metabolism under a wide range of environmental conditions. For further discussion of the sense in which bacteria qualify as minimal agents, see Fulda (2017). We thank the handling editor and an anonymous reviewer for drawing our attention to this important work.

14.
Barandiaran (2017) distinguishes between ‘monist’ and ‘pluralist’ approaches to autonomy. Monists accept only a single type of autonomy. Multicellular organisms are conceived of as second-order ensembles of autonomous systems. Ant colonies are in turn third-order ensembles (see, e.g., Bickhard, 2000; Christensen & Hooker, 2000). Pluralists allow for different types of autonomy, distinguishing the autonomy of the cell from that of the organism, or of behaviour (for an early statement, see Varela, 1979, 1991). For more recent exponents, see Barandiaran (2017) and Barandiaran and Moreno (2006). The pluralist makes distinctions between types of autonomy based on the identity of the system and the norms that arise in relation to this identity. The specific identity of the system defines the form that its structural coupling with the environment takes. We return to this last point in more detail later in this section, but we have nothing further to say about the debate between monists and pluralists.

15. The notion of behaviour settings originates in the work of the ecological psychologist Roget Barker (1968). For further discussion of Barker’s work, see Heft (2001, chs 7 & 8) and McGann (2014) for important suggestions about how to apply this notion within the enactive framework.

16. In the case of humans, affordances have what we have characterised as a socio material reality because they are realised in part through human practices. For further discussion, see Van Dijk & Rietveld (2016).

17. We thank an anonymous reviewer for emphasising this point to us. Important exceptions in the enactive literature are McGann (2014), De Haan et al. (2014) and Di Paolo et al. (2017). The former provides a careful analysis of the claim frequently made in the enactive literature that agent and environment stand in a relation of mutual interdependence (see, e.g., Thompson, 2007; Varela, Thompson, & Rosch, 1991). In doing so, McGann explicitly argues for closer engagement with ecological psychology by enactive cognitive scientists. De Haan et al. (2014) combined enactive cognitive science and ecological psychology to provide an account of the changes in the structure of experience in people being treated with deep brain stimulation for obsessions and compulsions. We have drawn extensively on Di Paolo et al. (2017) in developing the argument of section 2. The arguments of this section establish the relevance of their work to ecological psychology, in particular the notions of ‘micro-identities’ which we have explained in affordance-related terms.

18. What this criticism of Gibson misses, we suggest, is the way in which the concept of the ecological niche was for Gibson always to be understood in relation to the ‘way of life’ of a kind of animal, to what we have called a ‘form of life’ (Rietveld & Kiverstein, 2014).

19. Maturana and Varela (1980) charge representational theories of cognition with confusing and conflating the operational and relational domains in attempting to explain behaviour by reference to information-bearing internal states that properly speaking belong to the relational domain and are irrelevant to explaining the operational workings of living systems. However, such a line of argument depends on their internalist interpretation of biological autonomy we reject, so we have chosen not to follow them in embracing this distinction.

20. The latter concept is a development of Merleau-Ponty’s (1945/2002) concept of ‘*maximum prise*’. Hubert Dreyfus (2002) translated this term as ‘maximal grip’. We speak instead of a tendency towards an optimal grip to emphasise the way in which maximal grip always eludes an agent (Bruineberg & Rietveld, 2014; Rietveld, Denys, & Van Westen, in press). Instead, the agent experiences a deviation from an optimum (in its attunement to the environment), which the agent then tends to reduce. Unlike Dreyfus (2002), we do not limit ourselves to skills for acting pre-reflectively but also see reflective skills for imagination, planning and other examples of ‘representation-hungry cognition’ as part of the tendency towards grip on the environment.

21. For related line of argument, see Van Dijk and Withagen (2016).

22. We understand this reduction of disattunement in terms of *affordance-affordance related states of action-readiness* that help the individual tend towards this optimal grip (see Bruineberg & Rietveld, 2014). Perception is thus situated within a larger self-organising ‘brain-body-landscape of affordances’ system.

23. We take up this argument in detail in a companion piece to this article.

24. For further discussion of differences in agentive capacity among autonomous systems, see Di Paolo et al. (2017, §6.6).

25. See Gallagher (2017, §10.2) for a related suggestion. Also see Rucińska (2016) for a helpful treatment of play in terms of skilled engagement with affordances in an intersubjective context.
